# Objectively Measured Physical Activity, Sedentary Behavior, and Incident Fracture in Older Women: The OPACH Study

**DOI:** 10.1093/geroni/igab046.639

**Published:** 2021-12-17

**Authors:** Steve Nguyen, John Bellettiere, Michael LaMonte, Carolyn Crandall, Andrea LaCroix

**Affiliations:** 1 University of California, San Diego Herbert Wertheim School of Public Health and Human Longevity Science, La Jolla, California, United States; 2 University at Buffalo, Buffalo, New York, United States; 3 University of California, Los Angeles, Los Angeles, California, United States

## Abstract

Women aged 65 and older experience nearly three-fourths of the 2 million osteoporotic fractures annually in the US, yet whether accelerometer-measured volumes and intensities of physical activity and sedentary behavior (SB) are associated with reduced fracture risk is understudied. We investigated associations of accelerometer-measured light physical activity (LPA), moderate-to-vigorous physical activity (MVPA), sedentary time (ST), and mean sedentary bout duration (MBD) with incident clinical fractures (hip, vertebral, pelvis, lower leg, upper arm, forearm, and wrist) in the WHI OPACH cohort. Participants (N=6248; mean±SD age=78.6±6.7; 34% Black, 17% Hispanic) without prior hip fracture wore the ActiGraph GT3X+ for 7 days between May 2012-April 2014 and were followed through March 2020 for incident clinical fracture (N=711). Cox models estimated hazard ratios (HR) and 95% confidence intervals (CI), adjusting for age, race-ethnicity, education, alcohol, smoking, height, weight, falls history, RAND-36 physical function, diabetes, thiazide use, prescription osteoporotic therapy, and age at menopause. The HR(95% CI) across MVPA quartiles was 1.00(reference), 1.15(0.93-1.41), 0.90(0.72-1.13), and 0.79(0.61-1.02); p-trend=0.01. The HR(95% CI) for a one-interquartile range increment in MVPA (42 minutes/day) was 0.86(0.76-0.97). Associations were modified by prescription osteoporotic therapy [no: HR=0.77(0.66-0.89), yes: HR=1.03(0.85-1.25); p-interaction=0.01] and varied in magnitude by age[<80: HR=0.78(0.64-0.96), ≥80: HR=0.92(0.79-1.07); p-interaction=0.09], BMI [<30 kg/m2: HR=0.85(0.75-0.97), ≥30 kg/m2: HR=0.90(0.67-1.19); p-interaction=0.08], and race-ethnicity [Black: HR =0.63(0.44-0.89), Hispanic: HR =0.78(0.56-1.09), White: HR =0.92(0.80-1.06); p-interaction=0.16]. LPA, ST, or MBD were not associated with incident fractures. These data suggest that MVPA may reduce and not increase fracture risk and that LPA and SB do not increase fracture risk.

